# Quantifying Patient Demand for Orthopedics Care by Region Through Google Trends Analysis: Descriptive Epidemiology Study

**DOI:** 10.2196/63560

**Published:** 2025-01-31

**Authors:** Abram Qiu, Kristopher Meadows, Fei Ye, Osasu Iyawe, Kenneth Kenneth-Nwosa

**Affiliations:** 1 Department of Orthopaedics, University of Texas Health Science Center, San Antonio, TX, United States

**Keywords:** orthopedics, geographic factors, health care disparities, medical schools, internship and residency, epidemiology, public health informatics, physicians, assessment of health care needs, resource allocation

## Abstract

**Background:**

There is a growing gap between the supply of surgeons and the demand for orthopedic services in the United States.

**Objective:**

We analyzed publicly available online data to assess the correlation between the supply of orthopedic surgeons and patient demand across the United States. The geographic trends of this gap were assessed by using the relative demand index (RDI) to guide precision public health interventions such as resource allocation, residency program expansion, and workforce planning to specific regions.

**Methods:**

The data used were from the US Census Bureau, Association of American Medical Colleges (AAMC) through their 2024 Electronic Residency Application Service (ERAS) directory, AAMC State Physician Workforce Data Report, and Google Trends. We calculated the normalized relative search volume (RSV) and the RDI and compared them to the densities of orthopedic surgeons across the United States. We examined the disparities with the Spearman rank correlation coefficient.

**Results:**

The supply of orthopedic surgeons varied greatly across the United States, with a significantly higher demand for them in southern states (*P*=.02). The orthopedic surgeon concentration, normalized to the highest density, was the highest in Alaska (n=100), the District of Columbia (n=96), and Wyoming (n=72); and the lowest in Texas (n=0), Arkansas (n=6), and Oklahoma (n=64). The highest RDI values were observed in Utah (n=97), Florida (n=88), and Texas (n=83), while the lowest were observed in Alaska (n=0), the District of Columbia (n=5), and New Hampshire (n=7). The 7 states of Alaska, Maine, South Dakota, Wyoming, Montana, Delaware, and Idaho lacked orthopedic surgery residencies. In 2023, New York (n=19), Michigan (n=17), Ohio (n=17), Pennsylvania (n=16), and California (n=16) had the most residency programs. Demand and supply, represented by the RDI and orthopedic surgeon concentration, respectively, were strongly correlated negatively (ρ=−0.791, *P*<.001). States that were in the top quartile of residency programs (≥4 residency programs) exhibited a high demand for orthopedic surgeons (ρ=.6035, *P*=.02).

**Conclusions:**

This study showed that regional disparities in access to orthopedic care can be addressed by increasing orthopedic residencies. The study highlights the novel application of the RDI to mapping the regional need for orthopedics, and this map allows for better targeted resource allocation to expand orthopedic surgery training.

## Introduction

Orthopedic surgeries are among the most common medical procedures in the United States [1]. According to the American Academy of Orthopaedic Surgeons (AAOS), the number of orthopedic surgeons has not kept up with the need for orthopedic procedures. An AAOS fact sheet from March 2023 found that the current number of orthopedic surgeons must either double their total joint arthroplasty caseload or grow the number of surgeons by 10% every 5 years to meet the increasing demand [2]. This poses considerable challenges to the current state of health care, affecting the accessibility of services and increasing surgical workload amid physician burnout. We explored the geographic patterns of the supply of orthopedic surgeons and access to orthopedic services. The relative demand index (RDI) obtained in this study, derived from Google Trends data, provides a novel way to quantify the demand for orthopedic services considering the supply of residency-trained specialists across the United States. Unlike traditional measures of health care demand and supply, such as hospital utilization rates, census data, or claims data, Google Trends offers real-time insights into public interest and potential demand before it materializes as service utilization. The RDI leverages this data to provide a dynamic and location-specific perspective, highlighting regions where interest in orthopedic services may signal unmet health care needs. This approach also addresses the key limitations of traditional methods. Conventional metrics often rely on retrospective, static datasets that may not fully capture emerging trends in demand or geographic disparities. By contrast, the RDI uses Google Trends to reflect immediate, user-driven interest, enabling a more granular understanding of demand. Using the RDI, we highlighted underserved regions and offer recommendations for targeted public health interventions. Additionally, we aimed to examine the interplay between this trend of shortages and the orthopedic specialty interest as both a potential contributing factor to and a consequence of this phenomenon. This will yield better insight into these challenges and guide the use of resources for existing and new orthopedic surgery residencies to fill the gap between the need for orthopedic surgeons and the supply of their services.

## Methods

### Data Collection

We gathered data from publicly available sources. The state population totals from 2020 to 2022 were obtained from the United States Census Bureau [3]. The location of every accredited orthopedic surgery residency program in the United States was collected from the Association of American Medical Colleges (AAMC) through their 2024 Electronic Residency Application Service (ERAS) directory [4]. The data of the current physician supply, number of undergraduate medical education students, and number of graduate medical education (GME) residents and fellows in the United States were gathered from the most up-to-date 2021 AAMC State Physician Workforce Data Report [5]. Lastly, Google Trends was used to search for data of “Orthopedic surgeons” from 2004 to 2023 to determine the demand for orthopedic surgeons in the United States [6].

### Ethical Considerations

This study was conducted retrospectively and did not involve the use of any patient health record data. Therefore, it did not require assessment or approval by an institutional ethics review board.

### Analytical Metrics

To quantify the relative demand for orthopedic surgeons across the country, we first calculated the relative search volume (RSV) in each state by using the search volume data collected from Google. Google Trends reported the data on a state-by-state basis, so we divided the frequency of specific searches for “Orthopedic surgeons” in every state by the monthly total number of all other searches performed in the state and then obtained the average value across the 2004–2023 time span. This number was then normalized on a scale from 0 to 100 to obtain the RSV, a metric that allowed us to compare search volumes across states with different populations. The higher the normalized RSV, the higher the interest in the topic.

The RSV was then used to calculate the RDI, our quantitative metric for relative orthopedic surgeon demand. We divided the number of practicing orthopedic surgeons per state, obtained from the 2021 AAMC State Physician Workforce Data Report, by the United States census state population to calculate the approximate concentration of orthopedic surgeons per 10,000 individuals. The concentration of orthopedic surgeons per 10,000 individuals was normalized on a scale from 0 to 100, where 100 represented the greatest concentration. Normalization was performed through the minimum–maximum method. For each value, the difference between the minimum and maximum numbers in the dataset was divided by the range of the dataset. We were able to use this method to enhance readability and preserve relationships between the original values because no outliers were identified in our dataset based on interquartile ranges. All the calculations are shown below.


Relativesearchvolume=Frequencyofsearchesperstate/Totalmonthlysearchesperstate

Orthopedicsurgeonconcentration=(Orthopedicsurgeonsperstate/Statepopulation)×10,000

Relativedemandindex=Relativesearchvolume/Orthopedicsurgeonconcentration

The RSV was then divided by the concentration of orthopedic surgeons to obtain the RDI, also normalized on a scale from 0 to 100. The normalized RDI measures the relative demand for orthopedic surgeons by incorporating both their supply and demand; thus, a state with a normalized RDI of 100 has the highest relative demand and a state with a normalized RDI of 0 has the lowest demand. As a result, it also serves as a marker of supply; the states with a higher normalized RDI have a relative undersupply of orthopedic surgeons whereas the states with a lower normalized RDI have a relative oversupply.

For example, if State A has an RSV of 0.005 (0.5% of all searches) and a surgeon concentration of 1 surgeon per 10,000 people (0.0001), the RDI is 50. In contrast, if State B has the same RSV but a higher surgeon concentration of 1 surgeon per 5000 people (0.0002), the RDI is 25. Even with identical demand, the higher RDI for State A reflects a greater relative demand due to fewer surgeons available per person.

To highlight the geographical variation observed between the supply of and demand for orthopedic surgeons, we subtracted the concentration of orthopedic surgeons per 10,000 people from the normalized RDI. This yielded the difference in interest and concentration, a metric from 0 to 100 that describes the relationship between the supply of and the demand for orthopedic surgeons. The larger the difference, the greater the disparity between the search volume of orthopedic surgeons and the orthopedic surgeon concentration in the state, and conversely, there is less of a disparity when the difference is smaller.

### Statistical Analysis

Correlation analysis was run using Spearman rank correlation coefficient testing, which measures the strength and direction of association between two ranked variables. The statistical significance threshold was set as *P*<.05. Statistical analysis was performed with R software version 4.3.3 (R Foundation for Statistical Computing).

## Results

On preparing a graph of the normalized physician concentration versus the normalized RDI value across all states, we obtained a Spearman rank correlation coefficient of ρ=−0.791 (*P*<.001). This indicated that as the normalized RDI increased, the concentration of orthopedic surgeons decreased. On comparing the normalized RDI to the number of residency programs in the state, the coefficient was ρ=0.455 (*P*<.001). While it was a weaker association, it still indicated a high demand for orthopedic surgeons despite having many residency programs in the state. Alternatively, the large number of residency programs could be in response to the demand for orthopedics. Seven states had no residency programs for orthopedic surgery: Alaska, Maine, South Dakota, Wyoming, Montana, Delaware, and Idaho.

Our analysis of states below the 50th percentile of the RDI revealed an inverse relationship between the normalized RDI and physician concentration. This indicated that states with lower physician concentration exhibited a higher demand for orthopedic surgeons (ρ=−0.767, *P<*.001). Interestingly, within this subset of low-concentration states, a positive correlation was observed between the normalized RDI and the number of residency programs (ρ=0.510, *P=*.009). This suggested that increased residency training, or the number of residents who remain in the state they trained in, may not fully counteract the rising demand for orthopedic surgeons in these areas.

Furthermore, similar results were obtained when examining states with a normalized RDI exceeding the 50th percentile. The normalized RDI showed a negative correlation with physician concentration (ρ=−0.400, *P=*.044), again indicating a higher demand in areas with lower physician concentration. However, no statistically significant correlation was found between the normalized RDI and the number of residency programs in these high-demand states.

**Figure 1.**
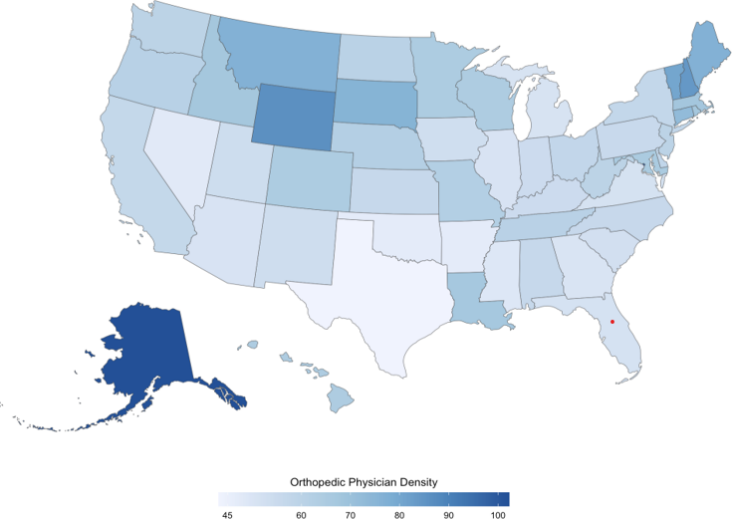
Orthopedic physician surgeons per 10,000 individuals across the United States.

As shown in Figure 1, Alaska (n=100), the District of Columbia (n=96), and Wyoming (n=72) had the greatest orthopedic surgeon concentration; and states like Texas (n=0), Arkansas (n=6), and Oklahoma (n=64) had the lowest concentration. Notably, Alaska and Wyoming were also among the 7 states without orthopedic surgery residency programs. also demonstrates that areas in the Midwest and Northeast of the country had a relatively higher concentration of orthopedic surgeons.

**Figure 2.**
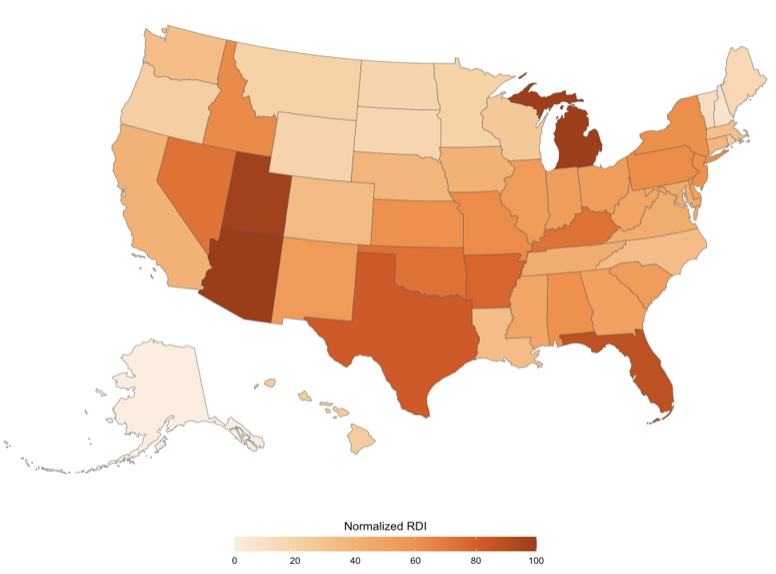
Normalized relative demand index (RDI) across the United States.

The states with the highest normalized RDI included Utah (n=97), Florida (n=88), and Texas (n=83), as highlighted in Figure 2. The territories with the lowest normalized RDI included Alaska (n=0), the District of Columbia (n=5), and New Hampshire (n=7). In contrast to the trend apparent with the orthopedic surgeon concentration, the states in the Midwest exhibited lower normalized RDI values, including states like South Dakota (n=0.17), North Dakota (n=18), Wyoming (n=19), Montana (n=20), and Minnesota (n=20). A few other states in the Northeast also had low normalized RDI values, such as Vermont (n=13) and Maine (n=16).

**Figure 3.**
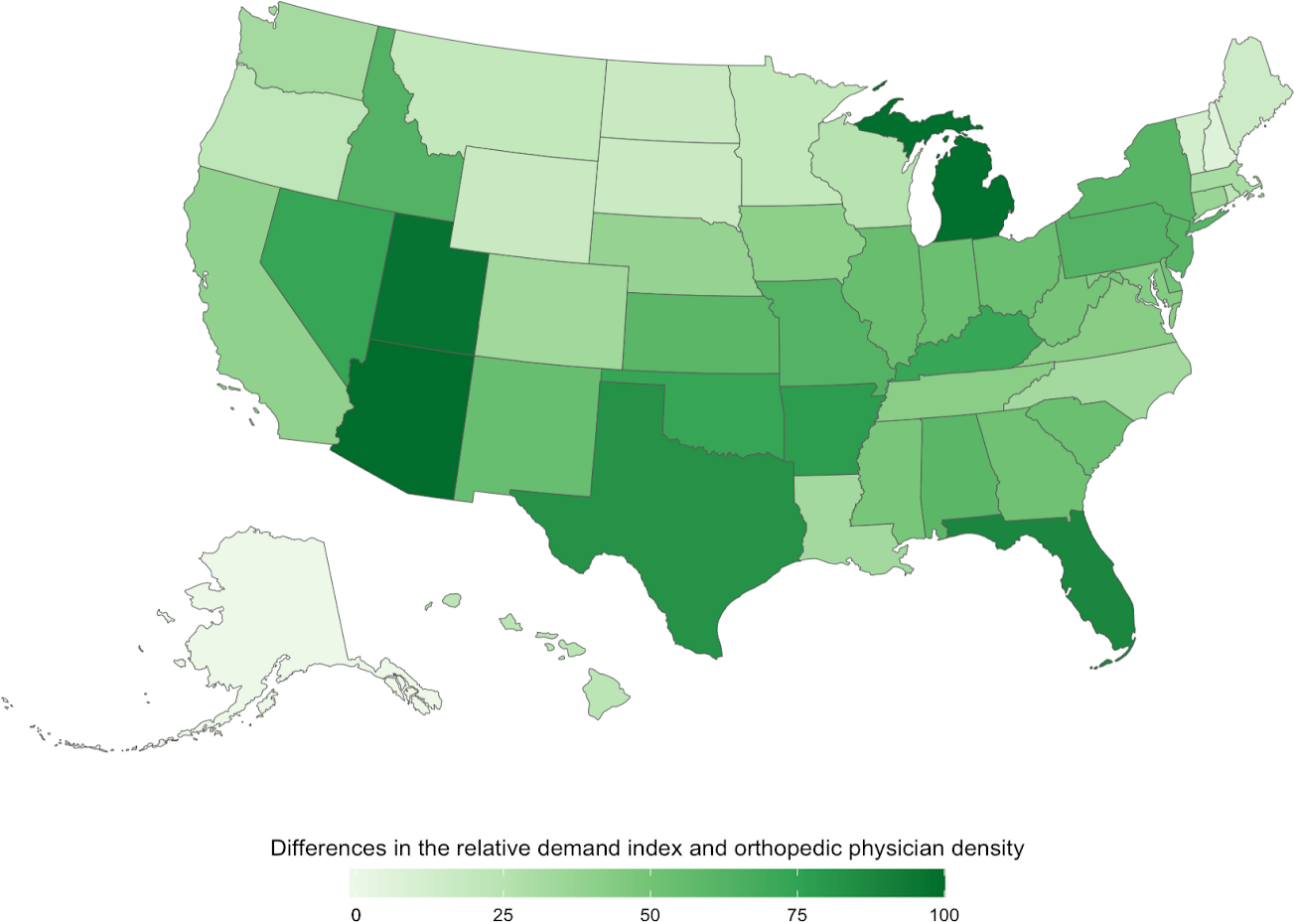
Differences in the relative demand index and orthopedic surgeon concentration across the United States.

The differences in the RDI and orthopedic surgeon concentration are mapped in Figure 3. The states with the greatest difference between public interest and the supply of orthopedic surgeons were Arizona (n=87), Michigan (n=86), and Texas (n=83). The states with the lowest differences included Alaska (n=−100), the District of Columbia (n=−90), and New Hampshire (n=−62).

Outside of Texas and Arizona, the country’s Southwestern region had the greatest disparity between the RDI and orthopedic surgeon concentration. Meanwhile, much of the results in the Southeast appeared relatively uniform, indicating similar low differences in the relative demand interest and orthopedic surgeon concentration across the region.

As shown in Table 1, we categorized the states based on their normalized RDI and orthopedic surgeon concentration. The values ≥50 were categorized as “high” normalized RDI, while those <50 were designated as “low” normalized RDI. No states had both a high normalized RDI and a high orthopedic surgeon concentration.

**Table 1.** States categorized by combinations of high or low normalized relative demand index (RDI) and orthopedic surgeon concentration.

Variable	High normalized RDI	Low normalized RDI
High orthopedic surgeon concentration	No states meet the criteria	Alaska District of Columbia Maine Montana New Hampshire South Dakota Vermont Wyoming
Low orthopedic surgeon concentration	Alabama Arizona Arkansas Delaware Florida Georgia Idaho Illinois Indiana Kansas Kentucky Michigan Missouri Nevada New Jersey New Mexico New York Ohio Oklahoma Pennsylvania South Carolina Texas Utah	California Colorado Connecticut Hawaii Iowa Louisiana Maryland Massachusetts Minnesota Mississippi Nebraska North Carolina North Dakota Oregon Rhode Island Tennessee Virginia Washington West Virginia Wisconsin

The high normalized RDI and low orthopedic surgeon concentration category excluded states bordering the Pacific Ocean or the Canadian border (except for Michigan). Instead, this category of states was skewed towards the central and southern regions of the United States. Additionally, while some Eastern states (Delaware, New Jersey, and New York) were present, the northernmost portion of the Eastern seaboard (Maine, Vermont, and New Hampshire) lacked representation. The lower supply of orthopedic surgeons and greater demand for them demonstrate that this category of states would be ideal locations for new residencies. They may also be a good place for new orthopedic surgery graduates to find employment.

In the low normalized RDI and high orthopedic surgeon concentration category, all the states were in the northern half of the United States, concentrated in the Northeast (Maine, New Hampshire, and Vermont) and Northwest (Alaska, Montana, and Wyoming).

The low normalized RDI and low orthopedic surgeon concentration category of states was the most geographically diverse. This group included states from across the country, with representation from the West Coast (California, Oregon, and Washington), East Coast (Connecticut, Maryland, Massachusetts, Rhode Island, and Virginia), Midwest (Iowa, Minnesota, Nebraska, and Wisconsin), and South (Louisiana, Mississippi, North Carolina, and Tennessee).

The data sources and the ways in which the data were used are shown in Table 2.

**Table 2.** Sources of data collected and the ways they were integrated into our analysis.

Data sources	Use of data
AAMC ERAS^a^ directory	Identified the number and location of all orthopedic surgery residency programs in the United States. Used the residency program data to compare to demand for orthopedic surgeons.
2021 AAMC State Physician Workforce Data	Provided the number of practicing orthopedic surgeons in the country. Used to quantify the concentration of orthopedic surgeons to create the relative demand index as well as the difference in interest and concentration of orthopedic surgeons.
United States Census Bureau	Provided state population totals during 2020‐2022. Used to calculate concentration in the RSV^b^ calculations.
Google Trends	Provided the volume of search data for “Orthopedic surgeons” during 2004‐2023. Used to calculate the RSV.

a AAMC ERAS: Association of American Medical Colleges through the Electronic Residency Application Service.

b RSV: relative search volume.

## Discussion

### Principal Findings and Comparison With Previous Studies

In this study, the observed relationship between the number of orthopedic residency programs and the normalized RDI is of interest. Certain states exhibited a scarcity of orthopedic residency programs and a limited demand for orthopedic services, such as South Dakota, North Dakota, Wyoming, Montana, and Minnesota. Conversely, a discernible trend emerged wherein states with a greater demand for orthopedic services showed a proportionate increase in the concentration of residency programs. This trend is found in other specialties as well. For example, in psychiatry, Pheister et al [7] found that 84.2% of residency program directors who started or expanded programs listed the shortage of local psychiatrists as the main driver.

We found a high orthopedic surgeon concentration and relatively low normalized RDI in the Midwest. This lower normalized RDI may signify that the demand for orthopedic surgeons is met with a greater concentration of such surgeons. As a region with a smaller concentration of metropolitan areas, finding that this market was saturated was particularly intriguing due to the well-documented trend of physicians preferring to practice in more urban regions. For example, in a systematic literature review, Cyr et al [8] concluded that specialists gravitate toward urbanized areas with more potential patients to support their practice.

Our approach has been validated by Ikpeze et al [9], Blau et al [10], and Akosman et al [11]. Combining Google Trends data and the number of orthopedic surgeons and residency programs from the AAMC allowed us to interpret the geographical distribution of demand. Ikpeze et al [9] examined the relationship between the orthopedic surgery applicant pool in the United States and Internet queries for orthopedic residency. They found that searches for “orthopedic residency” and “orthopedic salary” increased dramatically from 2004 to 2015. The National Resident Matching Program database also showed a consistent yearly increase in orthopedic surgery applicants. This positive correlation between an official database and Google Trends demonstrates its validity in gauging public interest in a topic [9].

Google Trends analysis has explored other specialties and topics outside of orthopedic surgery. Blau et al [10] looked at all searches for “plastic surgery” over 12 months, and similar to our study, used RSV and surgeons-per-capita value, or “surgical concentration.” Their Google RSV divided by this concentration yielded a “surgical demand index” for each state, which is similar to and validates our study’s normalized RDI. Their “surgical demand index” was the greatest in Wyoming, Oklahoma, and Arkansas; and the smallest in Oregon, Virginia, and Connecticut [10].

Akosman et al [11] performed a similar analysis for the ophthalmology specialty; they suggested that states with a higher normalized RDI may have an unmet medical need for ophthalmic care. The highest normalized RDI was found in South Dakota, Delaware, Michigan, and Arizona. The lowest normalized RDI was in Washington DC, Hawaii, Oregon, and Montana. In this study for the orthopedic specialty, we found the lowest normalized RDI to be in the District of Columbia, Alaska, and New Hampshire.

Career factors that may have influenced the geographical distribution include the supply of advanced medical facilities, renowned orthopedic institutions, research opportunities, and access to innovative technology. Additionally, lifestyle factors, including the overall quality of life, salary, cost of living, and recreational amenities in a particular state, are influential variables as well. It is difficult to objectively explain the drive to or away from certain states. The insight our data provides regarding the demand for orthopedic services can guide new orthopedic surgeons to places in the United States where they are needed. In addition, it could also inform where to establish new residencies, as well as how existing residency programs should invest in recruiting medical students to their state. The limitations to establishing such programs include high costs and complex requirements by the Accreditation Council for Graduate Medical Education (ACGME), which can be overcome by acquiring increased GME funding and partnering with hospitals, universities, and policymakers to reduce financial burden and facilitate meeting rigorous accreditation standards.

Future work may include combining orthopedic surgeon demand with the demand for specific orthopedic procedures. This would further develop our understanding of unmet orthopedic care needs in many states. For example, Cohen et al [12] measured the national public interest in the use of platelet-rich plasma for hip and knee osteoarthritis. In an analogous study with total knee arthroplasty and total hip arthroplasty, Cohen et al [13] also suggested that this data would be useful in making resource allocation decisions in states. Trends in the public interest may inform patient counseling, shared decision-making, and directions for future clinical research.

### Limitations

Google was the only search engine used in this study, and it is possible that data from other search engines may yield different results, potentially affecting the generalizability of our findings. Additionally, while this study provides insights into regional trends, the absence of complementary data, such as hiring rates or job postings, limits the ability to draw definitive conclusions about the supply-demand dynamics in orthopedic surgery. The use of general search terms, such as “orthopedic surgery,” introduces potential biases, including the influence of surges of unrelated searches that may not reflect genuine patient interest. For example, during emergencies, the volume of orthopedic surgeries and searches for orthopedic services may not be as high. Our dataset does not control for this. Furthermore, online search data may not accurately represent underserved populations or individuals with limited internet access, introducing inherent demographic and geographic biases. These limitations underscore the need for integrating online search data with traditional epidemiological and workforce datasets in future studies to achieve more nuanced and representative analyses.

### Conclusion

The supply of orthopedic surgeons varies greatly across the United States, with a higher demand for them in southern states. Demand and supply, represented by the RDI and orthopedic surgeon concentration, respectively, were strongly correlated negatively. States that were in the top quartile of residency programs (≥4 residency programs) exhibited a high demand for orthopedic surgeons. This study highlights the utility of the RDI as a scalable, data-driven tool for informing precision public health strategies. Beyond orthopedic care, the RDI framework could be adapted to other medical specialties, such as cardiology or general surgery, to identify underserved regions and guide resource allocation. For instance, the RDI can inform initiatives such as expanding residency programs, offering loan repayment or grant incentives, and deploying specialists to areas with high demand but limited supply. Additionally, if the RDI is correlated with specific procedures or conditions, such as knee surgeries in orthopedics or cardiac catheterizations in cardiology, it could be leveraged to target preventative public health initiatives. Examples include obesity reduction programs to decrease the need for joint replacements or smoking cessation campaigns to reduce cardiovascular disease prevalence. By integrating the RDI into broader public health frameworks, stakeholders can optimize care delivery, address disparities, and allocate resources more effectively across various health care settings.

## Abbreviations

AAMC: Association of American Medical Colleges
AAOS: American Academy of Orthopaedic Surgeons
ACGME: Accreditation Council for Graduate Medical Education
ERAS: Electronic Residency Application Service
GME: graduate medical education
RDI: relative demand index
RSV: relative search volume

## References

[1] Moldovan F, Moldovan L, Bataga T (2023). A comprehensive research on the prevalence and evolution trend of orthopedic surgeries in Romania. Healthcare (Basel).

[2] (2023). Fact sheet: orthopaedic surgeons will need to double total joint arthroplasty caseload to meet demand by 2050. American Academy of Orthopaedic Surgeons.

[3] State population totals and components of change: 2020-2022. United States Census Bureau.

[4] ERAS 2024 participating specialties & programs. Association of American Medical Colleges.

[5] 2021 state profiles. Association of American Medical Colleges.

[6] Orthopedic surgeons - explore. Google Trends.

[7] Pheister M, Cowley D, Sanders W (2022). Growing the psychiatry workforce through expansion or creation of residencies and fellowships: the results of a survey by the AADPRT workforce task force. Acad Psychiatry.

[8] Cyr ME, Etchin AG, Guthrie BJ, Benneyan JC (2019). Access to specialty healthcare in urban versus rural US populations: a systematic literature review. BMC Health Serv Res.

[9] Ikpeze TC, Mesfin A (2018). Interest in orthopedic surgery residency: a Google Trends analysis. J Surg Orthop Adv.

[10] Blau JA, Levites HA, Phillips BT, Hollenbeck ST (2020). Patient demand for plastic surgeons for every US state based on Google searches. JPRAS Open.

[11] Akosman S, Tran E, Rosenberg S, Pakhchanian H, Raiker R, Belyea DA (2024). Patient demand for ophthalmologists in the United States: a Google Trends analysis. Ophthalmic Epidemiol.

[12] Cohen SA, Zhuang T, Xiao M, Michaud JB, Amanatullah DF, Kamal RN (2021). Google Trends analysis shows increasing public interest in platelet-rich plasma injections for hip and knee osteoarthritis. J Arthroplasty.

[13] Cohen SA, Cohen LE, Tijerina JD (2021). Google Trends as a tool for evaluating public interest in total knee arthroplasty and total hip arthroplasty. J Clin Transl Res.

